# Effect of diets supplemented with linseed alone or combined with vitamin E and selenium or with plant extracts, on *Longissimus thoracis* transcriptome in growing-finishing Italian Large White pigs

**DOI:** 10.1186/s40104-018-0297-2

**Published:** 2018-11-20

**Authors:** Rubina Sirri, Marika Vitali, Paolo Zambonelli, Giulia Giannini, Martina Zappaterra, Domenico Pietro Lo Fiego, Dalal Sami, Roberta Davoli

**Affiliations:** 1 Interdepartmental Centre for Industrial Agrifood Research (CIRI- AGRO), University of Bologna, Via Quinto Bucci 336, I-47521 Cesena, Italy; 2 Department of Agricultural and Food Sciences (DISTAL), University of Bologna, Viale Fanin 46, I-40127 Bologna, Italy; 3 Department of Life Sciences, University of Modena and Reggio Emilia, Via G. Amendola 2, I-42122 Reggio Emilia, Italy; 4 Interdepartmental Research Centre for Agri-Food Biological Resources Improvement and Valorisation (BIOGEST-SITEIA), University of Modena and Reggio Emilia, P. le Europa, 1, I-42124 Reggio Emilia, Italy

**Keywords:** Diet supplementation, Functional analysis, Gene expression, Linseed, Muscle transcriptome, *n*-3 PUFA, Pigs, Plant extracts, Polyphenols, Vitamin E

## Abstract

**Background:**

Supplementing farm animals diet with functional ingredients may improve the nutritional quality of meat products. Diet composition has been also demonstrated to influence the gene expression with effect on biological processes and pathways. However, the knowledge on the effect of nutrients at the molecular level is scant. In particular, studies on the effects of antioxidants and polyphenols dietary supplementation have been investigated mainly in rodents, and only scarcely in farm animals so far. RNA-Seq with next-generation sequencing is increasingly the method of choice for studying changes in the transcriptome and it has been recently employed also in pig nutrigenomics studies to identify diet-induced changes in gene expression. The present study aimed to investigate the effect of diets enriched with functional ingredients (linseed, vitamin E and plant extracts) on the transcriptome of pig *Longissimus thoracis* to elucidate the role of these compounds in influencing genes involved in muscle physiology and metabolism compared to a standard diet.

**Results:**

Eight hundred ninety-three significant differentially expressed genes (DEGs) (FDR adjusted *P-*value ≤ 0.05) were detected by RNA-Seq analysis in the three diet comparisons (D2-D1, D3-D1, D4-D1). The functional analysis of DEGs showed that the diet enriched with *n*-3 PUFA from linseed (D2) mostly downregulated genes in pathways and biological processes (BPs) related to muscle development, contraction, and glycogen metabolism compared to the standard diet. The diet supplemented with linseed and vitamin E/Selenium (D3) showed to mostly downregulate genes linked to oxidative phosphorylation. Only few genes involved in extracellular matrix (ECM) organization were upregulated by the D3. Finally, the comparison D4-D1 showed that the diet supplemented with linseed and plant extracts (D4) upregulated the majority of genes compared to D1 that were involved in a complex network of pathways and BPs all connected by hub genes. In particular, *IGF2* was a hub gene connecting protein metabolism, ECM organization, immune system and lipid biosynthesis pathways.

**Conclusion:**

The supplementation of pig diet with *n*-3 PUFA from linseed, antioxidants and plant-derived polyphenols can influence the expression of a relevant number of genes in *Longissimus thoracis* muscle that are involved in a variety of biochemical pathways linked to muscle function and metabolism.

**Electronic supplementary material:**

The online version of this article (10.1186/s40104-018-0297-2) contains supplementary material, which is available to authorized users.

## Background

Several studies showed that supplementing farm animals diet with functional ingredients such as *n*-3 PUFA or antioxidants may improve the nutritional quality of meat products [1–6]. It is also known that diet composition may affect gene expression. However, the knowledge on the effect of nutrients at the molecular level is poorly known [7]. High-throughput technology such as RNA next-generation sequencing is presently an available method for nutrigenomics studies in order to identify diet-induced changes in the transcriptome [8]. In swine, few recent studies have used RNA-Seq to study the effect of diets supplemented with different fat sources or with *n*-3 and *n*-6 PUFA on the transcription profile of different tissues [7–9]. Other authors have studied in pigs the effects of plant-derived bioactive compounds, such as polyphenols, and synthetic antioxidants on gene expression by using microarray or qRT-PCR [10–14]. Both *n*-3 PUFA and antioxidants/polyphenols have been widely investigated since years for their positive role in human health and as nutraceuticals in several human diseases. These diet supplementations showed antioxidant and anti-inflammatory activity and positive effects against obesity and insulin-resistance [15–19]. In pigs, studies on diets supplemented with *n*-3 PUFA showed that they can influence the expression of genes involved in different biological processes (BPs) such as inflammatory response, fatty acid synthesis and oxidation [8, 9, 20–22], muscle development and differentiation, muscle protein metabolism [23] and glucose metabolism [21, 24]. However, studies on the effects of antioxidants and polyphenols dietary supplementation have been investigated mainly in rodents, and only scarcely in farm animals so far [10, 25]. The few studies in pigs reported that polyphenols are able to influence the expression of genes involved in lipid metabolism, inflammation and extracellular matrix (ECM) remodeling [10, 11]. However, the role of these compounds in healthy pigs and especially their effect on the pig transcriptome remains to be elucidated. For this reason, the aim of the present study was to analyse the differences between *Longissimus thoracis* muscle tissue transcriptome of pigs fed with three experimental diets with that of pigs treated with a standard diet, in order to investigate if the integration of extruded linseed, antioxidants and plant extracts can influence the expression of genes involved in muscle metabolism and physiology.

## Methods

All the experimental procedures performed in this study were in accordance with the national legislation and did not require special animal care authorizations according to the decision of the welfare committee of Consiglio per la Ricerca in agricoltura e l’analisi dell’economia agraria (CREA) taken the 14 September 2016 (Verbale 2) according to the Italian legislation, D. Lgs 4 Marzo 2014 n. 26 art. 2 punto F.

### Animals and sampling

A total of 48 Italian Large White pigs, 24 gilts and 24 barrows, were used for this study. The pigs were selected from a progeny of 258 piglets derived from 21 sows and 3 boars registered in the herd book of the Italian National Association of Pig Breeders (ANAS). All animals used in this study were kept according to the Council Rule (EC) No 1/2005 on the protection of animals during transport and related operations and amending Directives 64/432/EEC and 93/119/EC and Regulation (EC) No 1255/97.

After weaning, at the average live weight of 79.9 ± 5.8 kg, the pigs were divided into 4 groups of 12 animals each, balanced for weight, father and sex. Pigs after weaning were all fed a standard diet until the starting of the trial.

During the trial period, the pigs were fed four experimental diets: a standard diet for growing-finishing pigs (D1); a diet enriched with extruded linseed (source of *n*-3 PUFA) (D2); a diet enriched with extruded linseed, and vitamin E and selenium as antioxidants (D3); a diet enriched with extruded linseed and plant extract from grape-skin and oregano (source of polyphenols) (D4). Chemical composition of extruded linseed was characterized as follows: moisture (8%), crude fibre (25.0%), crude protein (20.2%), crude lipid (29.6%), and ash (3.0%). Fatty acid composition reported that content of α-linolenic acid was 54.7%, expressed in g/100 g of total fatty acids. *n*-3 PUFA content (g per 100 g of total fatty acids) was mainly constituted of α-linolenic acid and it was 5.2% in the control diet (D1) and 25.4% in D2, D3 and D4. The analytical total content of polyphenols in plant extract was 10.4 g/L for grape-skin extract and 3.9 g/L in oregano extract. Grape-skin extract was produced by Enocianina Fornaciari s.n.c. (Reggio Emilia, Italy) and oregano extract by Phenbiox s.r.l., (Bologna, Italy). Diets were adjusted within each experimental group according to pigs weight. During the first period, from an average weight of 79.9 ± 5.8 kg to 113.4 ± 10.6 kg, the amount of the supplied meal was calculated as 7.5% of the metabolic weight (1^st^ on Table 1). During the finishing period, from 113.4 ± 10.6 kg to the slaughter at an average weight of 150.5 ± 9.9 kg, the amount of the supplied meal was calculated as 8.5% of the metabolic weight (2^nd^ on Table 1). The detailed composition of the four experimental diets and the % of nutrients are reported in Table 1.

**Table 1:** Feed component and proximate composition of the experimental diets

		D1		D2		D3		D4	
		1^st^	2^nd^	1^st^	2^nd^	1^st^	2^nd^	1^st^	2^nd^
Ingredients									
Extruded linseed, %		-	-	5.00	5.00	5.00	5.00	5.00	5.00
Barley meal, %		85.50	91.00	80.50	86.60	80.30	86.40	80.50	86.60
Soya bean meal, %		11.00	5.50	11.00	5.00	11.00	5.00	11.00	5.00
*L*-Lysine, %		0.31	0.29	0.30	0.29	0.30	0.29	0.30	0.29
*DL*-Methionine, %		0.06	0.04	0.06	0.03	0.06	0.03	0.06	0.03
*L*-Threonine, %		0.05	0.04	0.05	0.03	0.05	0.03	0.05	0.03
Calcium carbonate, %		1.18	1.13	1.19	1.15	0.89	0.85	1.19	1.15
Dicalcium phosphate, %		1.00	1.10	1.00	1.00	1.00	1.00	1.00	1.00
Salt (NaCl), %		0.40	0.40	0.40	0.40	0.40	0.40	0.40	0.40
Vitamin/mineral pre-mix^1^, %		0.50	0.50	0.50	0.50	0.50	0.50	0.50	0.50
Vitamin E and Selenium pre-mix^2^, %		-	-	-	-	0.50	0.50	-	-
Plant extracts (Grape-skin + oregano), g/kg of feed		-	-	-	-	-	-	3.00+2.00	3.00+2.00
Proximate composition									
Digestible energy, kcal/kg		3189	3168	3255	3235	3248	3228	3255	3235
Crude protein, %		14.89	11.31	15.39	11.73	15.37	11.71	15.39	11.73
Crude fat, %		1.75	1.74	3.58	3.58	3.58	3.58	3.58	3.58
Crude fiber, %		4.33	4.20	4.62	4.48	4.61	4.47	4.62	4.48
Ca, %		0.80	0.79	0.82	0.79	0.82	0.79	0.82	0.79
P, %		0.54	0.54	0.55	0.53	0.55	0.53	0.55	0.53
Fatty acid composition, % (of total fatty acids)									
C14:0		0.47	0.39	0.25	0.21	0.25	0.22	0.26	0.22
C16:0		29.01	24.25	18.13	15.20	17.78	15.59	18.80	15.31
C16:1		0.49	0.34	0.17	0.15	0.17	0.17	0.02	0.15
C18:0		2.03	1.51	4.00	3.18	3.88	3.34	4.16	3.23
C18:1 *n-*9		14.92	13.50	20.60	18.12	20.24	18.45	21.29	18.26
C18:2 *n-*6		47.55	53.67	33.50	34.69	33.91	34.09	32.52	34.47
C18:3 *n-*3		4.77	5.70	22.83	28.02	23.25	27.73	22.38	27.95
C20:1		0.74	0.64	0.53	0.41	0.52	0.42	0.57	0.41

Legend: D1= standard diet for growing-finishing pigs; D2=standard diet supplemented with extruded linseed (source of *n*-3 PUFA); D3= standard diet supplemented with extruded linseed, vitamin E and selenium; D4= standard diet supplemented with extruded linseed and plant extracts (source of polyphenols). 1^st^ = feed administered from an average weight of 80 kg to 120 kg (growing period); 2^nd^ = feed administered from an average weight of 120 kg to slaughter (finishing period)

During the trial, one pig in the experimental group fed D4 died of natural causes. At the end of the trial, the animals were transported to a commercial abattoir. At the slaughterhouse, the pigs were electrical stunned and bled in a lying position in agreement with the Council Regulation (EC) No 1099/2009 on the protection of animals at the time of the killing. All slaughter procedures were monitored by the veterinary team appointed by the Italian Ministry of Health.

Slaughter was performed in two batches at an interval of 14 d one from the other. In order to have a similar weight for all the pigs at slaughter, the first batch included the six heaviest pigs of each group making a total of 24 animals. The remaining 6 pigs of each dietary group were then sent in the second batch after 14 d (23 pigs in the second batch as one died before the end of the experiment). Animals of each batch were loaded and transported together in the same truck. Groups were separated inside the truck and during all the pre-slaughter procedures to avoid stress response due to unfamiliar pig mixing. At the end of the slaughter line and before the carcass cooling, a sample from the *Longissimus thoracis* muscle of each pig was taken and immediately frozen in liquid nitrogen. Samples were then stored at -80°C until RNA extraction.

### RNA extraction, library preparation, sequencing

Total RNA was extracted using the standard RNA extraction method with TRIzol (Invitrogen, Carlsbad, CA, USA). Before use, RNA concentration in each sample was assayed with an ND-1000 spectrophotometer (NanoDrop Technologies) and its quality assessed with the Agilent 2100 Bioanalyzer with Agilent RNA 6000 Nano Kit (Agilent Technologies, Santa Clara, CA, USA).

Next-generation sequencing experiment was performed by the external service Genomix4life S.R.L. (Baronissi, Salerno, Italy). Indexed libraries were prepared from 1 μg of purified RNA from each sample with TruSeq Stranded mRNA (Illumina) Library Prep Kit according to the manufacturer’s instructions. Libraries were quantified using the Agilent 2100 Bioanalyzer (Agilent Technologies) and pooled (16 samples/pool for a total of 3 pools) such that each index-tagged sample was present in equimolar amounts, with a final concentration of the pooled samples of 2 nmol/L. The pooled samples were subject to cluster generation and sequencing using an Illumina HiSeq 2500 System (Illumina) in a 2×100 paired-end (RNA-seq) format loading the pool on a single lane. The raw sequence files generated are in FASTQ format.

### RNA-Seq data processing

RNA-seq data processing was performed by the external service MENTOTECH S.R.L. (Naples, Italy). The quality control of the raw reads was carried out using the FastQC tool (http://www.bioinformatics.babraham.ac.uk/projects/fastqc/), which generates a report for each sample read set. All reads were trimmed using the BBDuk software (https://jgi.doe.gov/data-and-tools/bbtools/) to eliminate Illumina adapters and bases with a quality Phred score lower than 25; only the reads with a length higher than 35 nucleotides were kept after trimming. The high-quality reads were aligned to the swine genome (Sscrofa11.1) using STAR aligner (version 2.5.2b, https://github.com/alexdobin/STAR/releases/tag/2.5.2b) [26]).

### Gene expression evaluation and differential expression assessment

These analyses were performed by the external service MENTOTECH S.R.L. (Naples, Italy). The program FeatureCounts implemented in Subread software (version 1.5.1) [27] was used to calculate gene expression values as raw fragment counts, followed by FPKM (Fragments Per Kilobase of transcript per Million mapped reads) calculation with EdgeR [28]. The identified genes were assessed for differential expression (DE) among diets: D2 vs. D1, D3 vs. D1, and D4 vs. D1 for a total of three comparisons. These comparisons were performed with NOISeq R/Bioc package [29], applying a TMM (Trimmed Mean of M-values) normalization, removing the genes with less than 1 CPM (counts per million) in all the samples and after applying the ARSyN (ASCA Removal of Systematic Noise for sequencing data) correction method using the dietary groups as experimental factors and father, sex and slaughter day as fixed factors. The posterior probabilities of differential expression were converted to FDR (False Discovery Rate) as indicated in the NOISeq manual. Differentially expressed genes were considered statistically significant according to FDR with an adjusted *P*-value ≤ 0.05.

### Validation by quantitative real-time PCR

The quantitative real-time PCR (qRT-PCR) standard curve method [30] was used to analyse the expression level of five genes selected among the differentially expressed genes (DEGs). The samples used for the validation came from the same RNA extraction used for RNA-Seq analysis. The synthesis of cDNA was performed from 1 μg of RNA using the ImProm-II™ Reverse Transcription System (Promega Corporation, Italy). qRT-PCR was performed on Rotor Gene™ 6000 (Corbett Life Science, Concorde, New South Wales) using 5 μL of SYBR® Premix Ex Taq™ (TAKARA Bio INC, Olsu, Shiga, Japan), 10 pmol of each primer, 2 μL of cDNA template diluted 1:10. Rotor Gene™ 6000 protocol was optimised using specific annealing temperatures for each primer couple (Additional file 1). qRT-PCR was performed using a two-step amplification constituted by a denaturation phase of 95°C for 5 s, followed by an annealing-extension phase at temperatures optimized per each primer couple for 20 s. Each cycle was repeated for 40 times. The variation coefficient (CV= standard deviation of the crossing points/average of the crossing points) of the replicated analysis for each sample (three in 2 different cycles of qRT-PCR) was set at 0.2 as maximum level accepted. Two housekeeping genes, beta-2-microglobulin (*B2M*) and hypoxanthine phosphoribosyltransferase 1 (*HPRT1*), were used to evaluate the expression level of the target genes. The expression levels of the five selected genes were then calculated using the standard curve methods, as described in Zappaterra et al. [31]. Pearson’s correlations were then calculated between qRT-PCR and RNA-Seq FPKM expression data for the five tested genes using the R software (R Core Team, 2017). The correlation coefficient (*r*) was considered significant with a *P* < 0.05.

### Functional classification of DEGs

For the analysis, only differentially expressed genes (DEGs) presenting a log_2_ fold change (log_2_FC) ≥ 0.30 or ≤ -0.30 and an FDR adjusted *P*-value ≤ 0.05 were considered (see Table 2 for number details). To have a more complete annotation than pig gene annotation, the *Homo sapiens* background was applied, so the gene IDs were converted to human gene IDs using BioMart – Ensembl (https://www.ensembl.org/biomart) prior to proceeding with the functional analysis. The functional analyses were performed on each diet comparison (D2-D1, D3-D1, D4-D1) separately.

**Table 2:** Up- and downregulated DEGs in each diet comparison before and after setting the log_2_FC cut-off

	D2-D1		D3-D1		D4-D1	
	All	Cut-off	All	Cut-off	All	Cut-off
Upregulated	6	6	13	13	91	73
Downregulated	469	151	291	63	23	20
All	475	157	304	76	114	93

The total number of DEGs (893 before and 326 after cut-off application) considers common genes among comparisons

### Cytoscape functional analysis

For functional enrichment analysis the Cytoscape v3.5.1 software (Institute for Genomics and Bioinformatics, Graz University of Technology, Graz, Austria) was used. First, a network of DEGs was built using the GeneMANIA plug-in [32] and then functional analysis was performed using the ClueGO plug-in [33]. ClueGO settings were applied in order to facilitate the identification and visualization of functional gene clusters. For each diet comparison, the ClueGO plug-in divided the significant DEGs into different functional groups having different *P*-values. Each functional group was indicative of a specific biological context and contained the pathways and BPs enriched by the diet in that specific biological context. The pathways and BPs of a functional group had different *P*-values and contained both up and downregulated DEGs. If upregulated DEGs were the majority, that pathway/biological process was assigned to cluster #1, thus considered upregulated; if the downregulated DEGs were the majority, that pathway/biological process was assigned to cluster #2, thus considered downregulated; finally if the percentage of both upregulated and downregulated DEGs in a pathway/biological process was ranging between 40-60%, the cluster was called None specific cluster. The log_2_FC values were selected as an attribute. The statistical method was set at right-sided hypergeometric distribution, and Bonferroni *P*-value correction was used. Minimum clustering was set at *P* ≤ 0.05 and minimum κ-score at 0.4. The biological process (BPs) ontology and KEGG and REACTOME pathways were used as databases for the functional analysis. Gene Ontology (GO) levels were set from 6 to 8, and a minimum number of genes per cluster was set at 5 (in case the number of DEGs in a cluster was minor than 5, the maximum number of available genes was inserted in that cluster). To graphically present the data obtained, REVIGO was employed to summarize the enriched GO terms and, when necessary, also the pathways were summarized by selecting the higher level in REACTOME pathway hierarchy (https://reactome.org/user/guide/pathway-browser). Subsequently, CytoHubba plug-in by using clustering coefficient statistical approach and CluePedia plug-in were applied to select and display in the figures the hub DEGs with the aim to visualize the interaction between the most significant DEGs and their related pathways and BPs. Only the pathways and BPs linked to these selected hub genes were chosen to graphically visualize the interaction between DEGs and GO Terms in the presented figures.

### DAVID functional analysis

The DAVID Functional Annotation Tool v. 6.8 (The Database for Annotation, Visualization, and Integrated Discovery: https://david.ncifcrf.gov/) was directed to identify Pathways from the Kyoto Encyclopedia of Genes and Genomes (KEGG) database and the Gene Ontology Biological Process [34]. The genes were uploaded as official gene symbol and human genome was selected as background. The *P*-value of the enrichment of the number of genes in BPs were evaluated using Benjamini's correction and *P* ≤ 0.05 was considered significant. The upregulated and downregulated DEGs were analyzed separately in each diet comparison.

## Results

### Sequencing output and identification of DEGs

**Fig. 1:**
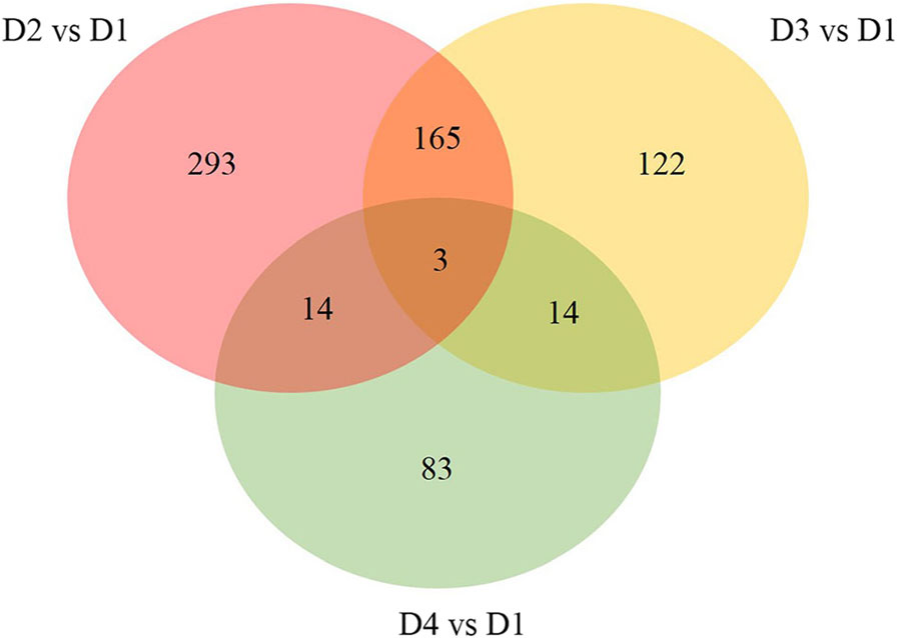
Venn diagram showing the distribution of the DEGs in the three diet comparisons

The total number of reads obtained are reported in Additional file 2. A total of 893 significant DEGs were detected by RNA-Seq analysis in the three diet comparisons (D2-D1, D3-D1, D4-D1). This number comprises also DEGs common to more than one comparison (the number of non-redundant unique genes was 695) (Fig. 1; Additional file 3). As the log_2_FC value of significant DEGs was quite low, a cut-off of the log_2_FC was selected in order to consider both the DEGs with the higher differences in expression and, to carry on the functional enriched analysis. After setting this cut-off the total number was reduced to 289 non-redundant DEGs, which were submitted to the functional analysis. In Table 2 is reported how the DEGs were distributed in each diet comparisons. The complete list of significant DEGs and the information about the log_2_FC and FDR adjusted *P*-value are reported in Additional file 3.

### Cytoscape functional analysis

**Fig. 2:**
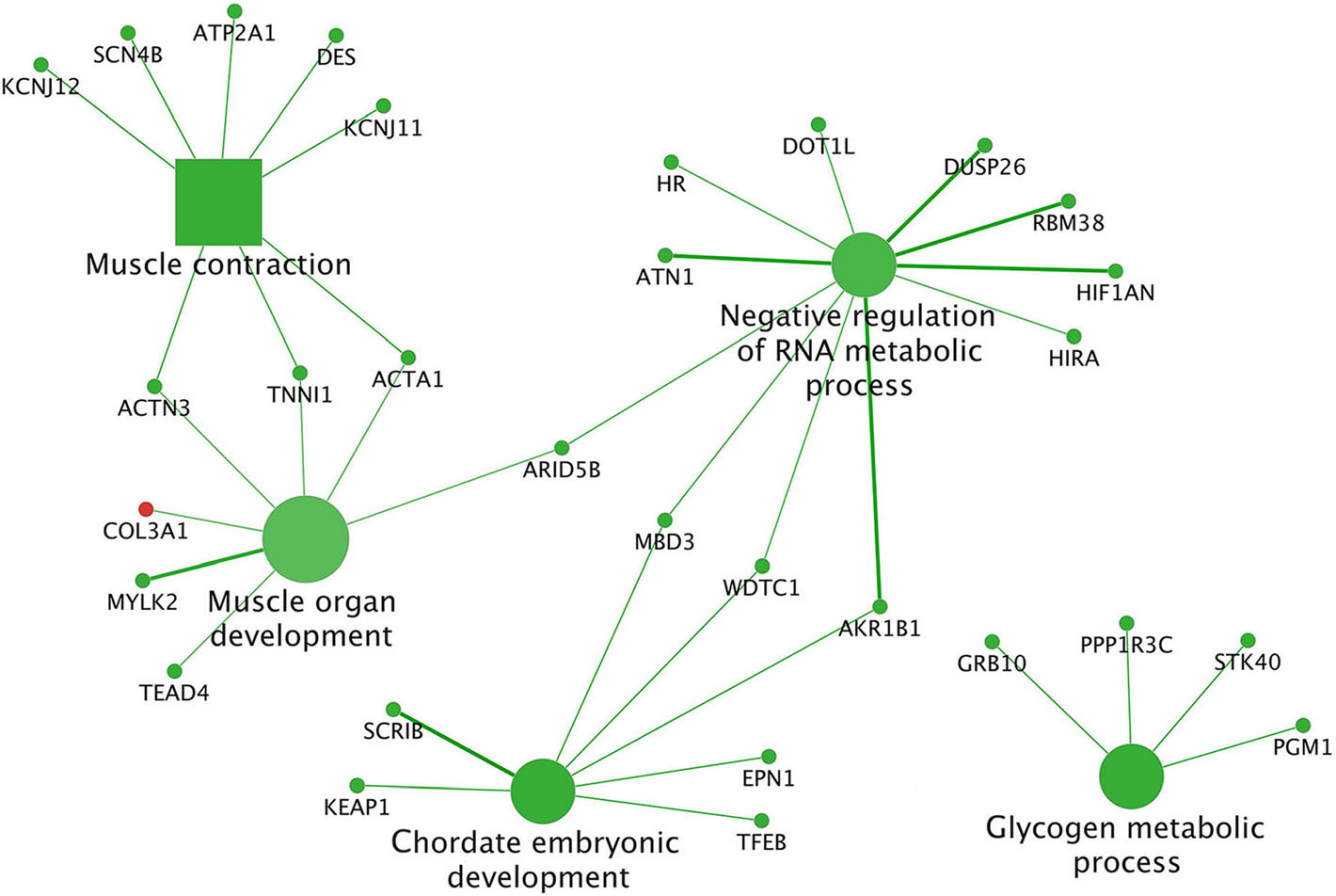
Functional analysis of D2-D1 comparison obtained using Cytoscape. Significant terms are graphically summarized using REVIGO. The figure shows the hub DEGs and the interactions with their related pathways and BPs. Legend: squares = pathways; circles = biological processes (BPs); shape size = according to the *P*-value of the term in its own group; red colour = upregulated (cluster #1) (only *COL3A1* is upregulated in this comparison); green colour = downregulated (cluster #2); interaction line thickness = according to kappa score value, represents the strength of the interactions, lighter colour corresponds to a lower strength while darker colour to a higher strength

In the comparison D2-D1, all the pathways and BPs were included in the cluster #2 (majority of the DEGs downregulated by the D2). This cluster comprised 5 different functional groups (Additional file 4 and Additional file 5). For each of these 5 functional groups it was identified the leading term, namely: "Negative regulation of RNA metabolic process" (*P* = 0.02), "Chordate embryonic development" (*P* = 0.02), "Muscle contraction" (*P* = 0.002), "Muscle organ development" (*P* = 0.004) and "Glycogen metabolic process" (*P* = 0.03). The complete list of all enriched terms in this comparison is reported in Additional file 4. The summarized pathways/BPs connected to the hub DEGs are displayed in Fig. 2. Biological terms related to human diseases were not displayed in this figure.

**Fig. 3:**
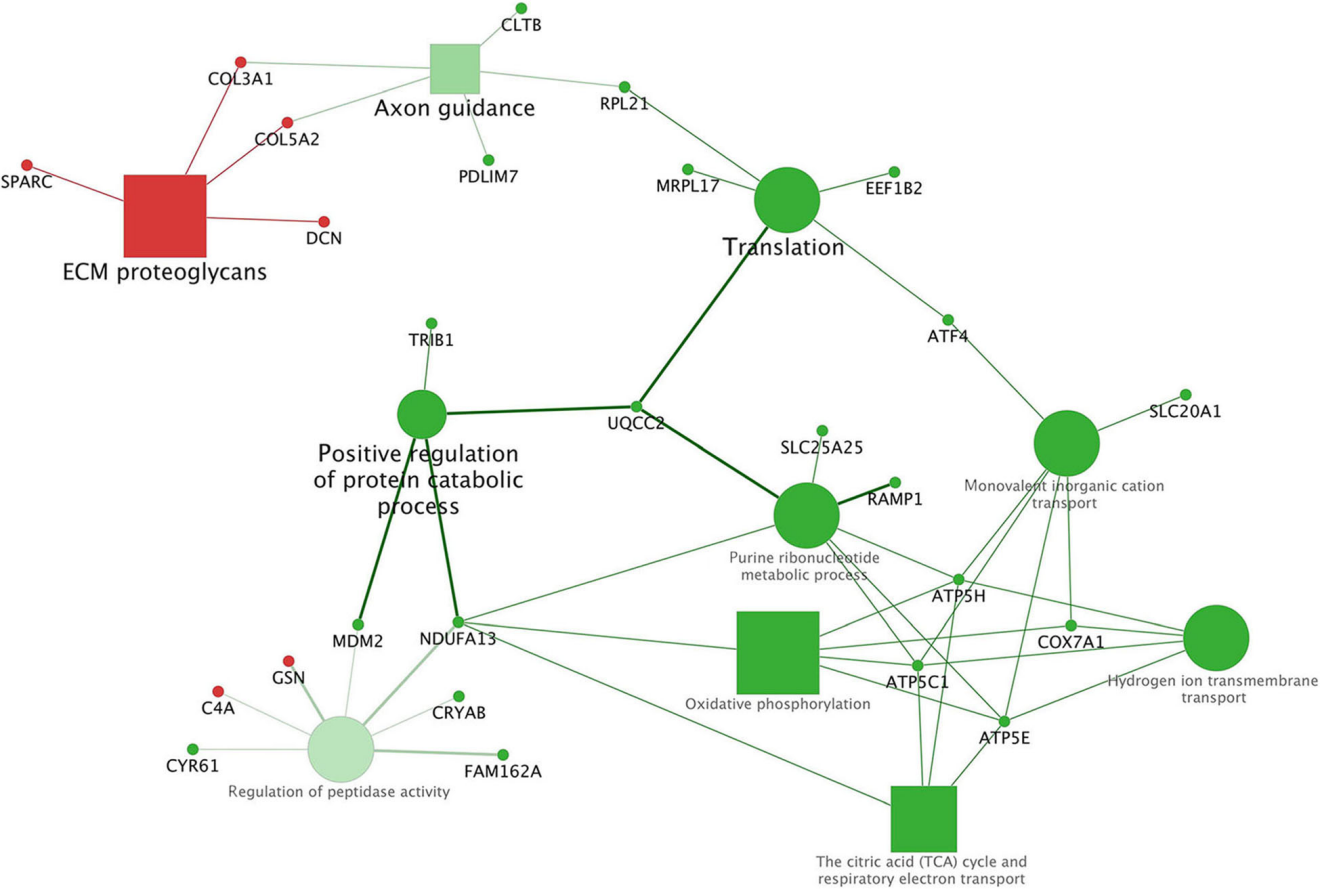
Functional analysis of DEGs found in the D3-D1 comparison obtained using Cytoscape. Significant terms are graphically summarized using REVIGO or manually according to the REACTOME database. The figure shows the hub DEGs and the interactions with their related pathways and BPs. Legend: squares = pathways; circles = biological processes (BPs); shape size = according to the *P*-value of the term in its own group; red colour = upregulated (cluster #1); green colour = downregulated (cluster #2); fill colour transparency = according to the percentage of genes belonging to the cluster; font size = according to the *P*-value of the term in its own group; interaction line thickness = according to kappa score value, represents the strength of the interactions, lighter colour corresponds to a lower strength while darker colour to a higher strength

Considering D3-D1 comparison, enriched pathways and BPs in the cluster #1 (majority of DEGs upregulated by the D3) were included in one functional group (Additional file 4 and Additional file 6) whose leading term was "ECM proteoglycans" (*P* = 0.00001). Instead, the enriched pathways and BPs included in the cluster #2 (majority of DEGs downregulated by the D3) was represented by 5 functional groups (Additional file 4). For each of these 5 functional groups it was identified the most significant pathway/biological process (leading term), namely: "Huntington’s disease" (*P* = 0.0003), "Regulation of cysteine-type endopeptidase activity involved in apoptotic process" (*P* = 0.0006), "Translation" (*P* = 0.004), "Axon guidance" (*P* = 0.008), "Positive regulation of protein catabolic process" (*P* = 0.01). The complete list of all enriched terms in this comparison is reported in Additional file 4. The summarized pathways/BPs connected to the hub DEGs are displayed in Fig. 3. Biological terms related to human diseases were not displayed in this figure.

**Fig. 4:**
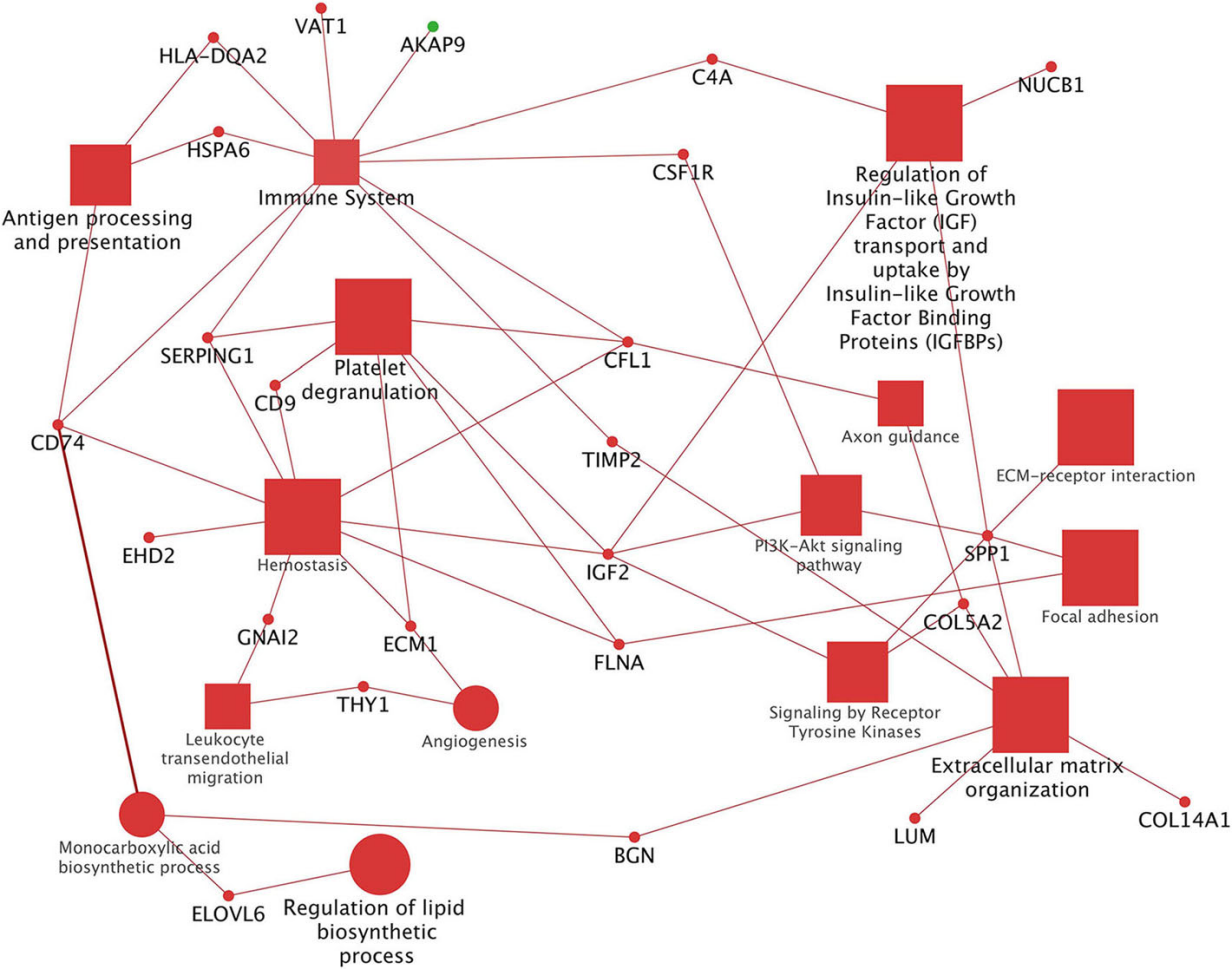
Functional analysis of DEGs found in the D4-D1 comparison obtained using Cytoscape. Significant terms are graphically summarized using REVIGO or manually according to the REACTOME database. The figure shows the hub DEGs and the interactions with their related pathways and BPs. Legend: squares = pathways; circles = biological processes (BPs); shape size = according to the *P*-value of the term in its own group; red colour = upregulated (cluster #1); green colour = downregulated (cluster #2) (only *AKAP9* is downregulated in this comparison); font size = according to the *P*-value of the term in its own group; interaction line thickness = according to kappa score value, represents the strength of the interactions, lighter colour corresponds to a lower strength while darker colour to a higher strength

In the comparison D4-D1, all the enriched pathways and BPs were included in the cluster #1 (majority of DEGs upregulated by the D4). This cluster comprised 13 different functional groups (Additional file 4 and Additional file 7). For each of these 13 groups it was identified the most significant pathway/biological process (leading term), namely: "Extracellular matrix organization" (*P* = 1.2E-17), "Platelet degranulation" (*P* = 5.3E-09), "Regulation of insulin-like growth factor (IGF) transport and uptake by insulin-like growth factor binding proteins (IGFBPs)" (*P* = 7.6E-08), "Regulation of cell morphogenesis" (*P* = 8.9E-07), "Regulation of cell migration" (*P* = 5.1E-06), "Regulation of peptidase activity" (*P* = 0.0002), "Regulation of lipid biosynthetic process" (*P* = 0.002), "Pertussis" (*P* = 0.003), "Antigen processing and presentation" (*P* = 0.003), "Regulation of phosphorylation" (*P* = 0.005), "Immune system" (*P* = 0.007), "Central nervous system development" (*P* = 0.04). The complete list of all enriched terms in this comparison is reported in Additional file 4. The summarized pathways/BPs connected to the hub DEGs are displayed in Fig. 4. Biological terms related to human diseases were not displayed in this figure.

### DAVID functional analysis

The use of DAVID software was carried-out in order to compare the results obtained by Cytoscape with another tool frequently used in literature to perform the functional analysis. The results obtained showed that DAVID included a lower number of DEGs in each pathway or BP than Cytoscape. Furthermore, DAVID did not show any significant pathway or BPs for the comparison D2-D1. On the other hand, the majority of the pathways and BPs found by Cytoscape in the comparison D3-D1 and D4-D1 were confirmed by DAVID for example, "ECM-receptor interaction" and "Oxidative phosphorylation" for the comparisons D3-D1, and "Fatty acid metabolism", "Extracellular matrix organization", "Focal adhesion", "Antigen processing and presentation" and "Platelet activation" for the comparison D4-D1. All the complete results from DAVID functional analysis of each diet comparison are reported in Additional file 8.

### Validation by quantitative real-time PCR

The validation of RNA-Seq results through qRT-PCR showed agreement between the gene expressions data obtained by the two methods. The five genes chosen for validation presented the same trend of expression and an overall good correlation between the two methods. Detailed results of Pearson’s correlation coefficients (*r*) and *P*-values are reported in Table 3.

**Table 3:** Pearson’s correlation coefficients (*r*) and *P*-values obtained comparing the expression level of genes tested by RNA-Seq and qRT-PCR

Gene	*r* coefficient	*P*-value	Diet comparison
*ELOVL6*	0.95	< 0.0001	D4-D1
*THBS1*	0.88	< 0.0001	D3-D1
*FASN*	0.87	< 0.0001	D4-D1
*CSRP3*	0.72	< 0.0001	D2-D1
*CSRP3*	0.67	0.0005	D4-D1
*SCD*	0.67	0.0004	D4-D1

## Discussion

The present study showed that the diets supplemented with extruded linseed (source of *n*-3 PUFA), antioxidants and plant extracts (rich in polyphenols) influence the transcription level of genes expressed in pig *Longissimus thoracis* muscle in comparison to the effect of a standard diet, with an impact on pathways and BPs. Looking at the number of DEGs reported in Table 2, the results showed that the supplementation with *n*-3 PUFA in D2 leads to downregulate many genes compared to D1. In fact, 151 DEGs out of 157 were underexpressed in D2 when compared to the control diet. Also in the comparison D3-D1, the supplementation with *n*-3 PUFA associated to vitamin E and selenium showed to reduce the expression of many genes in D3, as 63 DEGs out of 76 were downregulated compared to the control diet. By contrast, when *n*-3 PUFA supplementation is associated with plant derived-polyphenols in D4, 73 DEGs out of 93 were overexpressed by the D4 compared to the control diet. Therefore, we can suppose an effect of the *n*-3 PUFA supplementation alone in mainly downregulating the gene expression while by contrast, their combined supplementation with plant polyphenols leads to an upregulation of the majority of the genes.

The functional analysis performed using the two software evidenced that Cytoscape allows achieving a more complete and wider analysis of biological functions with respect to DAVID because DAVID does not consider upregulated and downregulated genes together.

The comparison between the diet supplemented only with *n*-3 PUFA (D2) compared to the standard diet (D1) showed a higher number of DEGs included in a lower number of pathways and BPs with respect to the other comparisons. The 151 genes downregulated in the D2 were involved in “Muscle contraction” (*ACTA1, ACTN3, ANXA6, ATP2A1, DES, KCNJ11, KCNJ12, SCN4B, TNNI1*) and “Muscle organ development” (*ARID5B, CSRP3, MYLK2, NEURL1, RXRA, SPEG, TEAD4, UQCC2*). Among these genes, *ARID5B* (*MRF2*) is an important transcription factor controlling myogenesis and its downregulation is reported by Tachtsis [35] to be linked to an arrest in myocytes differentiation. This gene was found in the present study as a hub gene connecting the “Muscle organ development” to the “Negative regulation of RNA metabolic process” BPs (Fig. 2) thus supposing a role of n-3 PUFA in suppressing muscle cell differentiation. In support of this, also other important transcriptional factors controlling muscle development like *MAPK6* and *MAPKAPK2* were downregulated in the D2 group. This is in agreement with different authors who reported an important role of *n*-3 PUFA in preserving muscle mass [36, 37]. In particular, Tachtsis et al. [35] showed that *n*-3 PUFA can preserve muscle mass by keeping cells in a quiescent state and preserving the satellite cells from their activation and differentiation. This mechanism is controlled by the MAPK signaling cascade in which *n*-3 PUFA have been proven to have a modulatory effect. In fact, Peng et al. [38] found that exposing proliferating C2C12 cells to DHA and EPA for 24 h decreased MAPK/ERK1/2 phosphorylation, preventing the progression of myoblasts from the G_1_ to S phase. These findings may confirm the results obtained in the present research and permit to hypothesize an involvement of *n*-3 PUFA supplementation in downregulating genes and transcriptional factors, *MAPK* and *ARID5B* (*MRF2*), involved in muscle differentiation with the aim to preserve muscle mass. A recent study in pigs by Ogłuszka et al. [9] also demonstrated that the supplementation of the diet with *n*-3 PUFA from linseed downregulated the expression of the majority of genes involved in muscle metabolism and functionality, including *MAPK1*, compared to a control diet. However, to date, there is a paucity of investigations on the effects of *n*-3 PUFAs on myogenesis and muscle health in vivo since most of the papers describe studies conducted in vitro [35, 39]. Furthermore, no studies were found in pigs, while human studies mainly investigated the effects of *n*-3 PUFA not in physiological conditions, making the comparison with the present results difficult.

The D2 compared to D1 showed also to downregulate genes involved in glucose and glycogen metabolism (*GRB10, PGM1, PPP1R3C, PYGM, STK40*). Among them, *PYGM* is known to encode a key enzyme of glycogenolysis and was downregulated in this study by the *n*-3 PUFA supplementation in D2. As some studies have shown that *n*-3 PUFA can improve glucose uptake and stimulate muscle glycogen synthesis [40, 41] in the present study we can hypothesize that *n*-3 PUFA supplementation suppressed genes involved in glycogenolysis thus supporting muscle glycogen storage.

Regarding the comparison D3-D1 the present research showed that the diet supplemented with *n*-3 PUFA, vitamin E and selenium (D3) downregulated the majority of the genes compared to D1. These genes, *ATP5C1, ATP5E, ATP5H, COX5B, COX7A1, NDUFA13* were involved in the “Oxidative phosphorylation” pathway and the mitochondrial electron transport chain. In fact, according to literature, the mitochondrial electron transport chain is one of the major cellular site of reactive oxygen species (ROS) generation and is considered important in the pathogenesis of neurodegenerative diseases such as Parkinson’s disease [42]. Moreover, the addition of *n*-3 PUFA has been demonstrated to enhance ROS production by mitochondria and cells [43]. In the present research, the addition of vitamin E and selenium to a diet supplemented with *n*-3 PUFA (D3) may prevent mitochondrial ROS formation by downregulating the genes involved in mitochondrial electron transport chain. Indeed, dietary antioxidants, including vitamin E, have been found to prevent the cells from this oxidative stress by reducing mitochondrial ROS production.

On the other hand, the D3-D1 comparison showed also an upregulation of some genes (*COL14A1, COL1A2, COL3A1, COL5A2, DCN, FN1* and *SPARC*) in D3 involved in the ECM organization and included in the term “ECM proteoglycans”. These results mainly agree with the few data reported in literature about an effect of antioxidants and *n*-3 PUFA in upregulating genes involved in ECM remodeling and organization. Villacorta et al. [44] suggested that α-tocopherol is able to induce in cell cultures the expression of connective tissue growth factor which in turn stimulates matrix production by inducing the expression of fibronectin, type I collagen and α5 integrin. Also in our study, we found overexpressed *FN1* and several genes of the *COL*s family, some of them as hub genes, thus supporting an effect of vitamin E in inducing the production of collagen and affecting the ECM organization. This effect of vitamin E may be intensified by the presence of *n*-3 PUFA in D3. In fact, Yoshino et al. [45] and Tachtsis et al. [35] reported that *n*-3 PUFA supplementation in human diet upregulated genes involved in ECM organization, thus modulating the function of skeletal muscle satellite cells, considering the reciprocal important relationship between ECM fibroblasts and muscle satellite cells. In this context, our results may suppose, according to the literature, an effect of both *n*-3 PUFA and vitamin E in positively stimulating the expression of genes coding for ECM proteins thus improving muscle function and cell-matrix interactions. However, the present research is the first one reporting the effect of a combined supplementation with vitamin E, selenium and *n*-3 PUFA on ECM genes in swine or other species.

The comparison D4-D1 resulted as the most complex one. In fact, this comparison involved a high number of pathways and BPs, all strongly connected through several hub genes (Fig. 4). This result leads to hypothesize an effect of *n*-3 PUFA and polyphenols in upregulating genes that have multiple biological functions and shared different physiological processes.

One of the hub genes that connected the majority of pathways and BPs was *IGF2*. This gene is involved in several metabolisms and functions, such as protein metabolism, glucose and lipid metabolism, cellular growth and immune system [46–48]. Furthermore, in pigs it has been evidenced that an increased expression of this gene can stimulate muscle growth [49]. In the present study, *IGF2* is involved in the pathway “Regulation of insulin-like growth factor (IGF) transport and uptake by insulin-like growth factor binding proteins (IGFBPs)”. The involvement of this gene in this pathway may lead to hypothesize that *IGF2* has a role in regulating the protein metabolism to maintain the structural integrity and physiology of muscle cells, also supported by the existing literature [50, 51]. The same pathway “Regulation of insulin-like growth factor (IGF) transport and uptake by insulin-like growth factor binding proteins (IGFBPs)” included also genes related to the ECM like *FN1, APP, SPP1* displaying a strong connection also to the “Extracellular matrix organization”. This means a possible association of *IGF2* with the ECM by binding to the integrins and playing a role in cell growth and signaling functions [52, 53]. The D4 positively stimulates several ECM genes involved in “Extracellular matrix organization”. The higher number of DEGs found in D4-D1 in this pathway were also observed in D3-D1 but in a lesser number, possibly indicating a stronger effect of D4 compared to D1 in influencing ECM genes. However, studies in literature reporting the role of polyphenols on the expression of genes encoding for ECM proteins are scant. Lin et al. [54] found that polyphenols may affect the expression of ECM genes in human peripheral blood cells and described the upregulation of genes encoding for collagen proteins, including *COL1A1* and *COL5A1*, like in our study. “Extracellular matrix organization” pathway showed also to contain *MMP2* and its inhibitor *TIMP2,* both upregulated by the D4. In literature, the co-expression of both MMPs and TIMPs proteins in skeletal muscle is found related to the regulation of muscle homeostasis and to maintain myofiber functional integrity in physiological conditions [55–57]. Unfortunately, no studies were found on the effect of polyphenols or *n*-3 PUFA on these genes under normal physiological conditions, suggesting that the comparison with the literature should be considered cautiously [16, 57, 58]. ECM genes are known to play an important role in cell signaling [59–61]. In particular, the genes of the *COLs* family, which are widely represented in our study, encode for proteins engaged in mechanical, structural and immune functions [62]. In fact, some genes involved in “Extracellular matrix organization” were also found in the “Immune system” pathway such as *APP, COL1A1, COL1A2, COL3A1, CTSB, FN1, MMP2* and *TIMP2*. This result is not surprising, because many ECM proteins play a role also in the tissue immune response [63]. Other genes involved in the immune system functional group and in the “Antigen processing and presentation” are the heat shock proteins *HSPA2* and *HSPA6*. In literature, polyphenols have been found to influence these genes in different ways by both increasing and decreasing the expression of *HSPs* [16]. However, *HSPs* genes are not only involved in cellular stress response but they also promote muscle development and functionality *via* myogenesis modulation or by maintaining the structural integrity of the proteins and signaling complexes [22, 64]. The two last above-cited pathways were found to be also related to the BP “Monocarboxylic acid biosynthetic process”. This BP is at the bottom of many others. The connector hub gene is *CD74* involved in stimulation of innate immune response and protein transport [65]. Through the hub gene *BGN*, involved in post-transduction regulation, DNA repair, ECM interaction and immunity [63], this pathway is in turn connected to “Extracellular matrix organization**”** (Fig. 4). This complex network leads to suppose a strong cross-talk among ECM, IGF2 signaling, immune system and lipid biosynthesis. In fact, the “Monocarboxylic acid biosynthetic process” is directly linked to the “Regulation of lipid biosynthetic process” through the hub gene *ELOVL6*, overexpressed in D4. As *IGF2* was evidenced by some authors to be linked to *ELOVL6* expression [47, 66] in the present study we can hypothesize a role of *IGF2* in triggering lipogenic gene expression with the aim to preserve membrane integrity of the muscle cells.

To the best of the authors’ knowledge, there is a lack of studies on the combined effect of polyphenols and *n*-3 PUFA on the expression of lipogenic genes in any animal species and tissues. Only the study from Kamei et al. [67] considers the effect of a diet supplemented with fat and maple syrup (rich in polyphenols) on mouse liver, evidencing similarly to our study an overexpression of *ELOVL6* and *SCD*. Torabi and DiMarco [68] reported that also polyphenols supplementation alone increased the expression of lipogenic genes in rat preadipocyte cultures. Moreover, considering that some studies reported also an upregulation of lipogenic genes following *n*-3 PUFA supplementation [8, 22] we hypothesize that the combined *n*-3 PUFA and polyphenols supplementation may trigger the expression of lipogenic genes.

To conclude, in D4-D1 comparison the functional analysis pointed out that the combined presence of polyphenols and *n*-3 PUFA in the diet was able to stimulate many biological processes and pathways involved in muscle functionality, development and homeostasis, probably through the stimulation of *IGF2* gene which influenced many processes including lipid biosynthesis, ECM organization and immune stimulation. However, due to the paucity of studies in vivo in this field, results should be confirmed with other studies and phenotypic measures.

## Conclusions

The overall results of the present study highlighted that the supplementation of pig diet with functional ingredients compared to a standard diet influenced the expression in *Longissimus thoracis* muscle of a relevant number of genes involved in a variety of biochemical pathways.

Diet supplemented with extruded linseed rich in *n*-3 PUFA (D2) overall reduced gene expression especially downregulating genes involved in the muscle development and contraction. Also the diet supplemented with linseed, vitamin E and selenium (D3) mainly reduced the gene expressions, particularly of genes involved in the oxidative phosphorylation, suggesting an antioxidant effect of vitamin E/selenium on *n*-3 PUFA-induced ROS production. Moreover, genes upregulated in D3 were linked to extracellular matrix organization, similarly to what was observed in D4. In addition in D4, genes linked to other important biological functions like lipid metabolism and immune system were also upregulated, leading to conclude that this diet may stimulate the overall muscle functionality. Due to the high number of biochemical functions stimulated and the high number of upregulated genes involved, this diet (D4) seems to have the major impact at the molecular level in skeletal muscle. However, the knowledge about the effect of these bioactive compounds on gene expression is limited in pigs and further investigation are deemed necessary.

## Additional files

Additional file 1: List of the genes used for RNA-Seq validation in this study. (DOCX 15 kb)

Additional file 2: Complete list of reads obtained from RNA-Seq analysis. (XLSX 14 kb)

Additional file 3: Complete list of differentially expressed genes in each diet comparison. (XLSX 146 kb)

Additional file 4: Full results from the functional analysis performed using the Cytoscape software. (XLSX 24 kb)

Additional file 5: Complete list of pathways and biological processes obtained from the functional analysis by Cytoscape in the D2-D1 comparison. Legend: squares = pathways; circles = biological processes (BPs); shape size = according to the *P*-value of the term in its own group; colour = terms belonging to the same functional group have the same colour; font size = according to the *P*-value of the term in its own group; interaction line thickness = according to Kappa Score value, represents the strength of the interactions, lighter colour corresponds to a lower strength while darker colour to a higher strength. (JPEG 170 kb)

Additional file 6: Complete list of pathways and biological processes obtained from the functional analysis by Cytoscape in the D3-D1 comparison. Legend: squares = pathways; circles = biological processes (BPs); shape size = according to the *P*-value of the term in its own group; colour = terms belonging to the same functional group have the same colour; font size = according to the *P*-value of the term in its own group; interaction line thickness = according to kappa score value, represents the strength of the interactions, lighter colour corresponds to a lower strength while darker colour to a higher strength. (JPEG 241 kb)

Additional file 7: Complete list of pathways and biological processes obtained from the functional analysis by Cytoscape in the D4-D1 comparison. Legend: squares = pathways; circles = biological processes (BPs); shape size = according to the *P*-value of the term in its own group; colour = terms belonging to the same functional group have the same colour; font size = according to the *P*-value of the term in its own group; interaction line thickness = according to kappa score value, represents the strength of the interactions, lighter colour corresponds to a lower strength while darker colour to a higher strength. (JPEG 241 kb)

Additional file 8: Full results from the functional analysis performed using the DAVID web resource. The term UP refers to genes upregulated in the first diet cited of each comparison, while DOWN refers to genes downregulated in the first diet cited of each comparison. (XLSX 29 kb)

## Abbreviations

ACTA1: Actin, alpha 1, skeletal muscle
ACTN3: Actinin alpha 3
ANXA6: Annexin A6
APP: Amyloid beta precursor protein
ARID5B (MRF2): AT-rich interaction domain 5B
ARSyN: ASCA removal of systematic noise for sequencing data
ATP2A1: ATPase sarcoplasmic/endoplasmic reticulum Ca^2+^ transporting 1
ATP5C1: ATP synthase F1 subunit gamma 1
ATP5E: ATP synthase F1 subunit epsilon
ATP5H: ATP synthase peripheral stalk subunit d
B2M: Beta-2-microglobulin
BGN: Biglycan
BP: Biological process
CD74: CD74 molecule
cDNA: Complementary DNA
COL14A1: Collagen type XIV alpha 1 chain
COL1A1: Collagen type I alpha 1 chain
COL1A2: Collagen type I alpha 2 chain
COL3A1: Collagen type III alpha 1 chain
COL5A1: Collagen type V alpha 1 chain
COL5A2: Collagen type V alpha 2 chain
COLs: Collagens gene family
COX5B: Mitochondrial cytochrome c oxidase subunit Vb
COX7A1: Cytochrome c oxidase polypeptide VIIa-muscle/heart
CPM: Counts per million
CSRP3: Cysteine and glycine-rich protein 3
CTSB: Cathepsin B
D: Diet
DCN: Decorin
DEG: Differentially expressed gene
DES: Desmin
DHA: Docosahexaenoic acid
DNA: Deoxyribonucleic acid
ECM: Extracellular matrix
ELOVL6: Fatty acid elongase 6
EPA: Eicosapentaenoic acid
ERK1/2: Extracellular signal-regulated protein kinases 1 and 2
FA: Fatty acid
FASN: Fatty acid synthase
FDR: False discovery rate
FN1: Fibronectin 1
FPKM: Fragments per kilobase of transcript per million mapped reads
GO: Gene Ontology
GRB10: Growth factor receptor bound protein 10
HKG: Housekeeping gene
HPRT1: Hypoxanthine phosphoribosyltransferase 1
HSPA2: Heat shock protein family A (HSP70) member 2
HSPA6: Heat shock protein family A (HSP70) member 6
HSPs: Heat shock proteins family
IGF2: Insulin-like growth factor 2
IGFBPs: Insulin-like growth factor binding proteins
KCNJ11: Potassium voltage-gated channel subfamily J member 11
KCNJ12: Potassium voltage-gated channel subfamily J member 12
log_2_FC: log_2_-based fold change
MAPK: Mitogen-activated protein kinase
MAPK1: Mitogen-activated protein kinase 1
MAPK6: Mitogen-activated protein kinase 6
MAPKAPK2: Mitogen-activated protein kinase-activated protein kinase 2
MMP2: Matrix metallopeptidase 2
MMPs: Matrix metallopeptidases family
mRNA: Messenger ribonucleic acid
MYLK2: Myosin light chain kinase 2
NDUFA13: NADH:ubiquinone oxidoreductase subunit A13
NEURL1: Neuralized E3 ubiquitin protein ligase 1
PCR: Polymerase chain reaction
PGM1: Phosphoglucomutase 1
PPP1R3C: Protein phosphatase 1 regulatory subunit 3C
PUFA: Polyunsaturated fatty acid
PYGM: Glycogen phosphorylase, muscle associated
qRT-PCR: Quantitative real-time polymerase chain reaction
RNA: Ribonucleic acid
RNA-Seq: Ribonucleic acid sequencing
ROS: Reactive oxygen species
RXRA: Retinoid X receptor alpha
SCD: Stearoyl-CoA desaturase
SCN4B: Sodium voltage-gated channel beta subunit 4
SPARC: Secreted protein acidic and cysteine-rich
SPEG: SPEG complex locus
SPP1: Secreted phosphoprotein 1
STK40: Serine/threonine kinase 40
TEAD4: TEA domain transcription factor 4
THBS1: Thrombospondin 1
TIMP2: TIMP metallopeptidase inhibitor 2
TIMPs: Tissue inhibitor of metalloproteinase gene family
TMM: Trimmed mean of M-values
TNNI1: Troponin I1, slow skeletal type
UQCC2: Ubiquinol-cytochrome c reductase complex assembly factor 2
