# Supplementary material for: Effect of diets supplemented with linseed alone or combined with vitamin E and selenium or with plant extracts, on Longissimus thoracis transcriptome in growing-finishing Italian Large White pigs
Source: J Anim Sci Biotechnol. 2018 Nov 20;9:81. doi: 10.1186/s40104-018-0297-2 (PMC6245756; doi:10.1186/s40104-018-0297-2)
Supplement: Supplementary file 1 — List of the genes used for RNA-Seq validation in this study. (DOCX 15 kb) [file 40104_2018_297_MOESM1_ESM.docx]

**ADDITIONAL FILE 1**

**Table S1.** List of the genes used for RNA-seq validation in this study. TM = Annealing temperature.

| **Gene name** | **Acronym** | **Primers** | **TM (°C)** |
| --- | --- | --- | --- |
| Elongation Of Long Chain  Fatty Acids isoform 1 | *ELOVL6* | F AGCAGTTCAACGAGAACGAAGCC | 66 |
|  |  | R TGCCGACCGCCAAAGATAAAG |  |
| Fatty Acid Synthase | *FASN* | F ATGCCGAAGGGACCGGCTAT | 67 |
|  |  | R CATTGAGGATGGTGGCGTAT |  |
| Stearoyl-CoA Desaturase | *SCD* | F CCGGGAGAATATCCTGGTTT | 66 |
|  |  | R GGTAGTTGTGGAAGCCCTCA |  |
| Thrombospondin 1 | *THBS1* | F GCGTTGGTGATGAGACAGAA | 64 |
|  |  | R CAAAGCAAGGATTGGACAGG |  |
| Cysteine and glycine-rich protein 3 | *CSRP3* | F ACGCAGAAGAAATCCAGTGC | 66 |
|  |  | R TGCTACCGTGGTGCTGTCTA |  |
| Beta-2-Microglobulin | *B2M* | F CCTTCTGGTCCACACTGAGT | 66 |
|  |  | R TCCCACTTAACTATCTTGGGCT |  |
| Hypoxanthine Phosphoribosyltransferase 1 | *HPRT1* | F CCCAGCGTCGTGATTAGTGA | 66 |
|  |  | R CCTTTTCCAAATCCTCGGCA |  |
