# Supplementary figures and images for: Effect of diets supplemented with linseed alone or combined with vitamin E and selenium or with plant extracts, on Longissimus thoracis transcriptome in growing-finishing Italian Large White pigs
Source: J Anim Sci Biotechnol. 2018 Nov 20;9:81. doi: 10.1186/s40104-018-0297-2 (PMC6245756; doi:10.1186/s40104-018-0297-2)

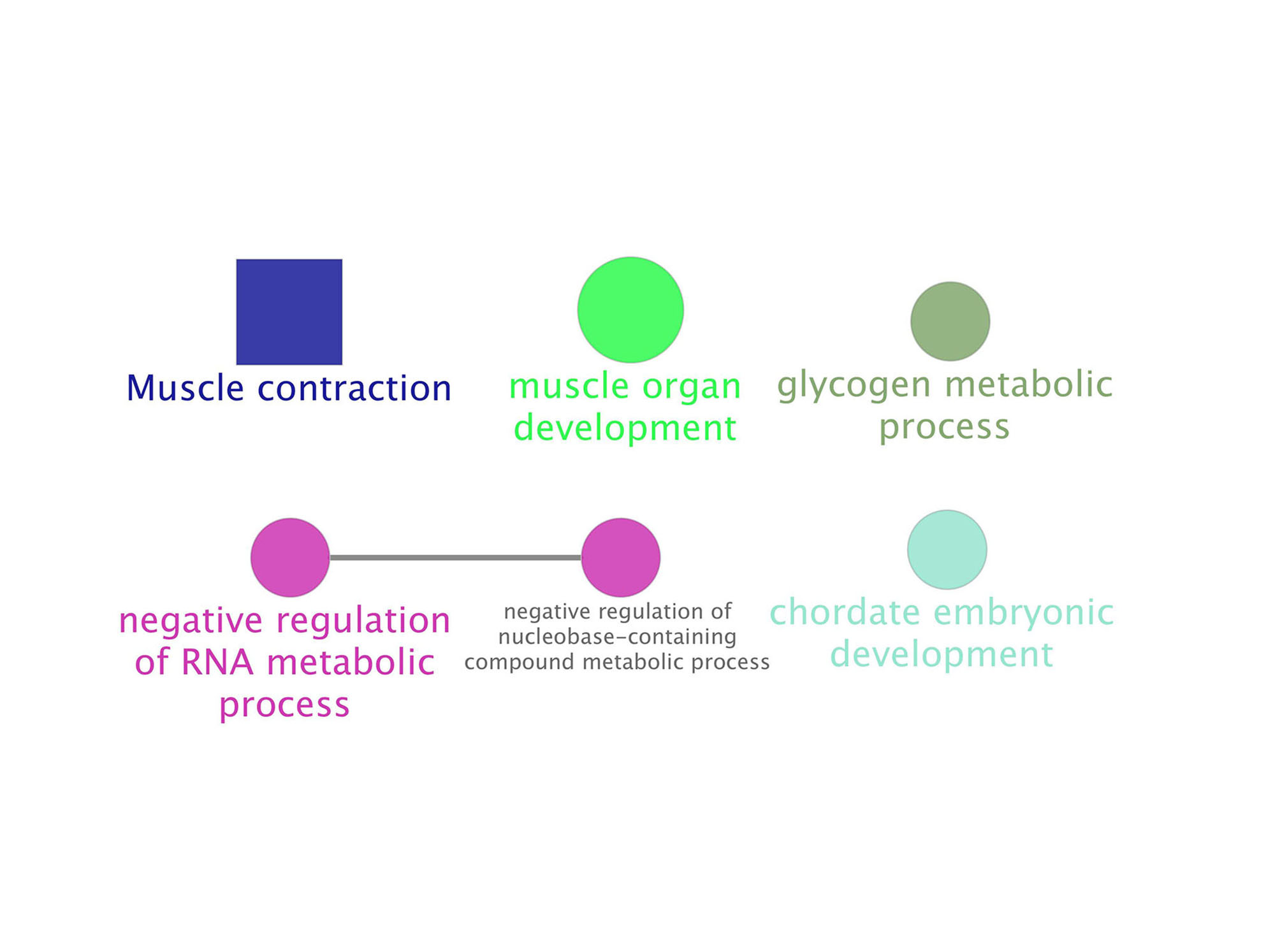

Supplement: Supplementary file 5 — Complete list of pathways and biological processes obtained from the functional analysis by Cytoscape in the D2-D1 comparison. Legend: squares = pathways; circles = biological processes (BPs); shape size = according to the P-value of the term in its own group; colour = terms belonging to the same functional group have the same colour; font size = according to the P-value of the term in its own group; interaction line thickness = according to Kappa Score value, represents the strength of the interactions, lighter colour corresponds to a lower strength while darker colour to a higher strength. (JPEG 170 kb) [file 40104_2018_297_MOESM5_ESM.jpeg]

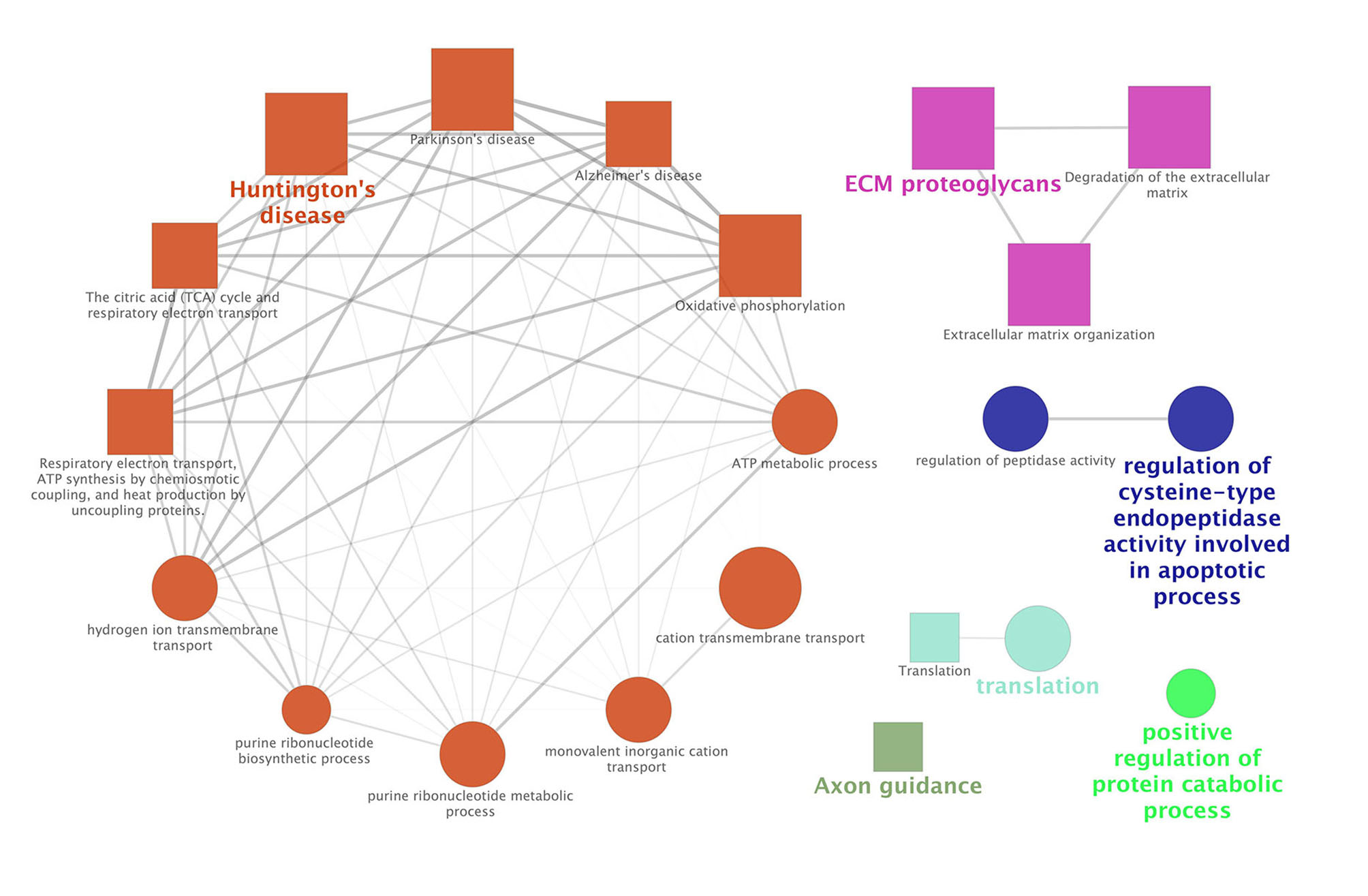

Supplement: Supplementary file 6 — Complete list of pathways and biological processes obtained from the functional analysis by Cytoscape in the D3-D1 comparison. Legend: squares = pathways; circles = biological processes (BPs); shape size = according to the P-value of the term in its own group; colour = terms belonging to the same functional group have the same colour; font size = according to the P-value of the term in its own group; interaction line thickness = according to kappa score value, represents the strength of the interactions, lighter colour corresponds to a lower strength while darker colour to a higher strength. (JPEG 241 kb) [file 40104_2018_297_MOESM6_ESM.jpeg]

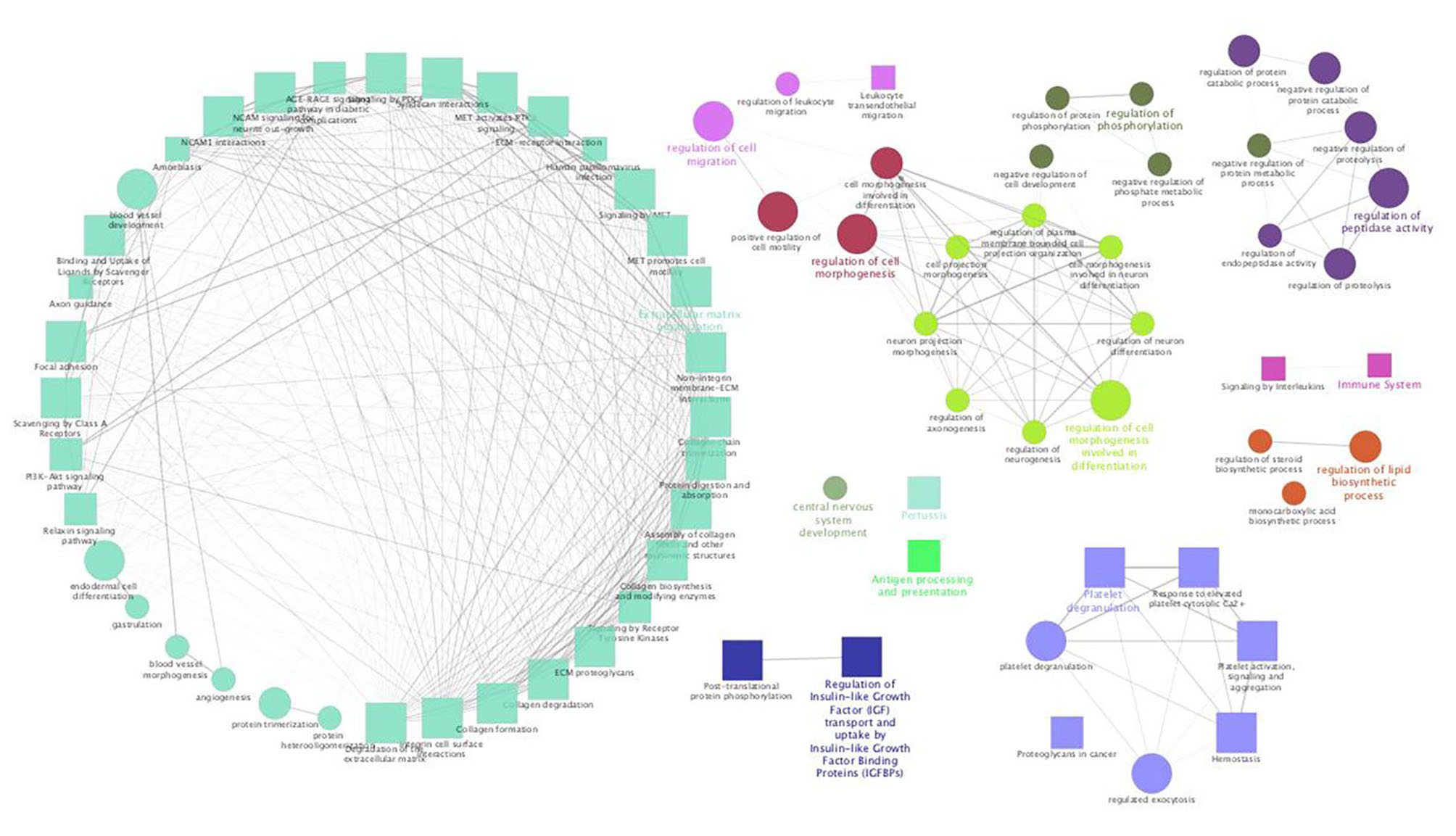

Supplement: Supplementary file 7 — Complete list of pathways and biological processes obtained from the functional analysis by Cytoscape in the D4-D1 comparison. Legend: squares = pathways; circles = biological processes (BPs); shape size = according to the P-value of the term in its own group; colour = terms belonging to the same functional group have the same colour; font size = according to the P-value of the term in its own group; interaction line thickness = according to kappa score value, represents the strength of the interactions, lighter colour corresponds to a lower strength while darker colour to a higher strength. (JPEG 241 kb) [file 40104_2018_297_MOESM7_ESM.jpeg]
